# A precise oral glucose loading ^18^F-FDG PET myocardial metabolic imaging protocol in coronary artery disease patients complicated by diabetes mellitus: an exploratory prospective cohort study

**DOI:** 10.3389/fmed.2026.1899226

**Published:** 2026-07-10

**Authors:** Liang Yin, Yangyang Lin, Jianlan Yue, Zhichun Lin, Hongtao Sun

**Affiliations:** 1 Department of Nuclear Medicine, Characteristic Medical Center of Chinese People’s Armed Police Forces, Pingjin Hospital, Tianjin, China; 2 Tianjin Children’s Hospital (Children's Hospital, Tianjin University), Tianjin Key Laboratory of Birth Defects for Prevention and Treatment, Tianjin, China; 3 Tianjin Key Laboratory of Neurotrauma Repair, Institute of Neurotrauma Repair, Characteristic Medical Center of People’s Armed Police Forces, Pingjin Hospital, Tianjin, China

**Keywords:** ^18^F-FDG, coronary artery disease, diabetes mellitus, myocardial viability, PET

## Abstract

**Objectives:**

We evaluated the feasibility of using a precise oral glucose loading combined with subcutaneous insulin administration protocol for ^18^F-FDG PET myocardial metabolic imaging in patients with coronary artery disease (CAD) complicated by diabetes mellitus (DM).

**Methods:**

179 consecutive CAD patients complicated by DM were enrolled. Oral glucose was given at variable doses based on baseline blood glucose, and subcutaneous insulin was injected at adjusted doses according to post-load glucose levels before FDG injection. SPECT myocardial perfusion imaging and PET myocardial metabolic imaging were performed using a one-day protocol. The image quality and adverse reaction data were analyzed.

**Results:**

Among 179 patients, FDG image quality was scored as excellent in 57.5%, very good in 31.8%, good in 7.8%, fair in 1.7% and non-diagnostic in 1.1%. When patients were stratified by blood glucose level at time of FDG injection >7.77 mmol/L, 5.55–7.77 mmol/L and <5.55 mmol/L, the image quality were excellent in 43.1, 60.4 and 74.1%, very good in 43.1, 30.7 and 14.8%, good in 9.8, 5.9 and 11.1%, fair in 3.9, 1.0% and 0, and non-diagnostic in 0, 2.0% and 0, respectively. Patients were divided into insulin therapy group and oral medication group, the image quality were excellent in 50.7 and 62.5%, very good in 33.3 and 30.8%, good in 10.7 and 5.8%, fair in 2.7 and 1.0%, and non-diagnostic in 2.7% and 0, respectively. Patients with blood glucose at time of FDG injection >7.77 mmol/L had significantly lower odds of excellent image quality (*p* = 0.021) than those with 5.55–7.77 mmol/L or <5.55 mmol/L, while no significant difference was observed between the latter two groups (*p* = 0.262). 47 patients (26.3%) exhibited completely viable myocardium, 89 patients (49.7%) had partially viable myocardium, and 43 patients (24.0%) showed nonviable myocardium. None of the patients had severe adverse reactions.

**Conclusion:**

The precise oral glucose loading protocol prior to ^18^F-FDG injection is feasible for clinical application.

## 1 Introduction

The global morbidity and mortality of coronary artery disease (CAD) are rising worldwide, with prevalence doubling from 271 million cases in 1990 to 523 million in 2019 (1). Coronary revascularization serves as an effective therapeutic strategy for CAD. Nevertheless, patients lacking sufficient viable myocardium may not benefit from the procedure and could even be at risk of surgical mortality. Accordingly, preoperative assessment of viable myocardium quantity constitutes one of the core determinants for formulating individualized treatment strategies. Imaging techniques such as echocardiography, cardiac magnetic resonance (CMR), and cardiac fluorodeoxyglucose positron emission tomography (^18^F-FDG PET) are mainstream modalities applied for myocardial viability assessment. Stress echocardiography boasts high specificity, yet it exhibits significant inter-observer variability; meanwhile, its diagnostic accuracy declines markedly in patients with severe left ventricular dysfunction (2). CMR is contraindicated in patients with implanted metallic devices (3); additionally, gadolinium-based contrast agents are prohibited for patients with renal insufficiency whose estimated glomerular filtration rate (eGFR) is below 30 mL/min/1.73 m^2^.

The combination of FDG PET myocardial metabolic imaging (MMI) and myocardial perfusion imaging (MPI) is regarded as the gold standard for assessing myocardial viability (4). Accurate identification of viable myocardium is achieved by comparing the level of MMI with the status of MPI. A concordant decrease in both MPI and MMI is classified as a perfusion-metabolism match, which denotes nonviable myocardium. A more marked reduction in MPI relative to MMI is defined as a perfusion-metabolism mismatch, indicating viable myocardium. Schinkel et al. reported that the weighted mean sensitivity and specificity of cardiac FDG PET reach 92 and 63%, respectively (5). To obtain adequate myocardial ^18^F-FDG uptake for optimal image quality, metabolic preparation prior to scanning is commonly relatively complex and time-consuming, especially for patients with diabetes mellitus (DM) (6). However, CAD often coexists with DM patients (7); accordingly, further intensive studies on MMI are highly necessary for such patients. The protocol combining oral glucose loading with insulin administration is widely preferred due to its simplicity and practicality. In the guidelines of the American Society of Nuclear Cardiology (ASNC), the standard oral glucose loading protocol typically uses glucose doses ranging from 25 g to 100 g, followed by administration of a certain dosage of intravenous insulin according to post-load blood glucose levels (8). Nevertheless, this protocol fails to incorporate individualized glucose doses tailored to varying baseline blood glucose, nor does it differentiate glucose doses between diabetic and non-diabetic patients. In addition, studies focusing on exploring precise oral glucose dosage regimens for diabetic patients undergoing myocardial metabolic imaging remain scarce. Furthermore, no published literature has reported the application of subcutaneous insulin injection in ^18^F-FDG PET MMI to date. Accordingly, the present study aimed to evaluate the feasibility and practicality of a precise and individualized oral glucose loading combined with subcutaneous insulin injection protocol for ^18^F-FDG PET MMI in CAD patients complicated by DM.

## 2 Materials and methods

### 2.1 Study population

This was a prospective, exploratory feasibility cohort study conducted at Characteristic Medical Center of Chinese People’s Armed Police Forces, Pingjin Hospital, between December 2017 and December 2025. A total of 179 consecutive patients with CAD complicated by DM (75 received insulin therapy and 104 were treated with oral medication only), who were assessed by ^99^ᵐTc-methoxyisobutylisonitrile (MIBI) SPECT rest MPI combined with ^18^F-FDG PET gated MMI for viable myocardium, were enrolled in the study. Inclusion criteria were as follows: patients with myocardial infarction or ischemic cardiomyopathy complicated by diabetes, as diagnosed in accordance with the American Diabetes Association guidelines (9). Patients with abnormal hepatic and renal function, inflammatory diseases including infective endocarditis and pericarditis were excluded. The study protocol adhered to the tenets of the Declaration of Helsinki and was approved by the Institutional Review Board of Characteristic Medical Center of Chinese People’s Armed Police Forces, Pingjin Hospital. Written informed consent was obtained from all individual participants included in the study. Participants were informed about the purpose of the study, the procedures involved, potential risks, and their right to withdraw at any time without consequence.

### 2.2 Metabolic preparation protocol

All patients were instructed to fast for a minimum of 6 h before PET examination. Patients receiving oral hypoglycemic agents were told to withhold their morning doses of anti-diabetic medications. The metabolic preparation procedure was carried out by a trained experienced nurse in a dedicated preparation room.

First, baseline blood glucose (All blood glucose measurements in this study were capillary blood glucose) was measured at the initiation of preparation. When baseline blood glucose were less than or equal to 6.0 mmol/L, from >6.0 mmol/L to ≤7.0 mmol/L, from >7.0 mmol/L to ≤8.0 mmol/L, from >8.0 mmol/L to ≤9.0 mmol/L, from >9.0 mmol/L to ≤10.0 mmol/L, from >10.0 mmol/L to ≤11.0 mmol/L and >11.0 mmol/L, then 50 g, 40 g, 30 g, 20 g, 15 g, 10 g and 5 g of 50% dextrose in 50 mL of water were orally, respectively (Table 1). Post-load blood glucose was rechecked after approximately 50-min (range, 45 ~ 60 min) interval to determine the optimal dosing of regular insulin (Wuxi Yushou Medical Appliances Co., Ltd) administration. If the post-load blood glucose were greater than or equal to 13 mmol/L, from 12 mmol/L to less than 13 mmol/L, from 11 mmol/L to less than 12 mmol/L, from 10 mmol/L to less than 11 mmol/L, from 9 mmol/L to less than 10 mmol/L, from 8 mmol/L to less than 9 mmol/L, from 7 mmol/L to less than 8 mmol/L, then 12 IU, 10 IU, 8 IU, 7 IU, 6 IU, 4 IU and 2 IU insulin was administered subcutaneously, respectively (Table 1). Fifty minutes later (range: 45–60 min), blood glucose was measured again. If the blood glucose reduction failed to reach the preset target, subcutaneous insulin injection was repeated according to the updated blood glucose value, followed by another blood glucose test after a further 45–60 min interval. The primary target was to lower blood glucose to <7.77 mmol/L prior to ^18^F-FDG injection. For a small subset of patients who cannot tolerate prolonged metabolic preparation period (MPP, it was defined as the period between oral 50% dextrose and ^18^F-FDG injection) resulting from repeated insulin injections, the minimum blood glucose decline relative to the post-load blood glucose peak shall be ≥1.11 mmol/L (Must received at least 1 subcutaneous insulin injection). Subsequently, approximately 326 ± 78 MBq (range: 185–410 MBq) of ^18^F-FDG was administered via intravenous bolus injection immediately.

**Table 1:** Precise oral glucose dose and subcutaneous insulin dose protocol.

Oral glucose protocol		Subcutaneous insulin injection protocol	
Baseline blood glucose level (mmol/L)	Oral glucose dose (g)	Post-load blood glucose levels (mmol/L)	Subcutaneous insulin dose (regular insulin, U)
< = 6	50	> = 13	12
6 ~ 7	40	12 ~ 13	10
7 ~ 8	30	11 ~ 12	8
8 ~ 9	20	10 ~ 11	7
9 ~ 10	15	9 ~ 10	6
10 ~ 11	10	8 ~ 9	4
>11	5	7 ~ 8	2

If symptomatic or asymptomatic hypoglycemia (blood glucose <3.9 mmol/L) occurred after insulin injection, the protocol allowed for appropriate oral glucose solution promptly to restore normal glycemia. If patients developed palpitation, dizziness, sweating, hunger or other hypoglycemic symptoms after insulin administration while blood glucose remained within normal limits, regular food intake was permitted 15 min after ^18^F-FDG injection. SPECT-MPI and gated PET-MMI were performed using a one-day protocol. ^99m^Tc-MIBI was injected immediately after oral glucose administration, and MPI imaging was completed during the MPP.

### 2.3 Images acquisition

Images of cardiac SPECT-MPI were acquired 60–90 min after injection of 740 MBq ^99m^Tc-MIBI using a Millennium SPECT scanner (GE Healthcare, USA) equipped with a dual-head gamma camera and high-resolution collimators. A 64 × 64 acquisition matrix and a zoom factor of 1.33 were applied for image acquisition. The images were reconstructed via attenuation-weighted ordered subset expectation maximization iterative reconstruction (8 subsets, 4 iterations).

Images of cardiac FDG PET-MMI were acquired 50 ~ 75 min after FDG injection using a Discovery ST16 PET/CT scanner (GE Healthcare, USA) with patients placed in a supine position and their arms raised bilaterally. The CT data (11 eff mA, 140 kV, helical thickness 5 mm, pitch 1.375) was performed both for attenuation correction and anatomic localization. Cardiac emission images were acquired in a single bed position with a zoom factor of 2 over 10 min in 3D mode, with 8 frames acquired per R-R interval. Acquired data were reconstructed via an iterative method (Iterations: 2; Subsets: 21). Reconstruction was performed on a 128 × 128 matrix, yielding a pixel size of 2.34 mm and slice thickness of 2.34 mm. The acquired datasets were reconstructed using Myovation or ECToolbox application software and displayed as short-axis, horizontal and vertical long-axis slices.

### 2.4 Cardiac ^18^F-FDG PET image analysis

**Figure 1:**
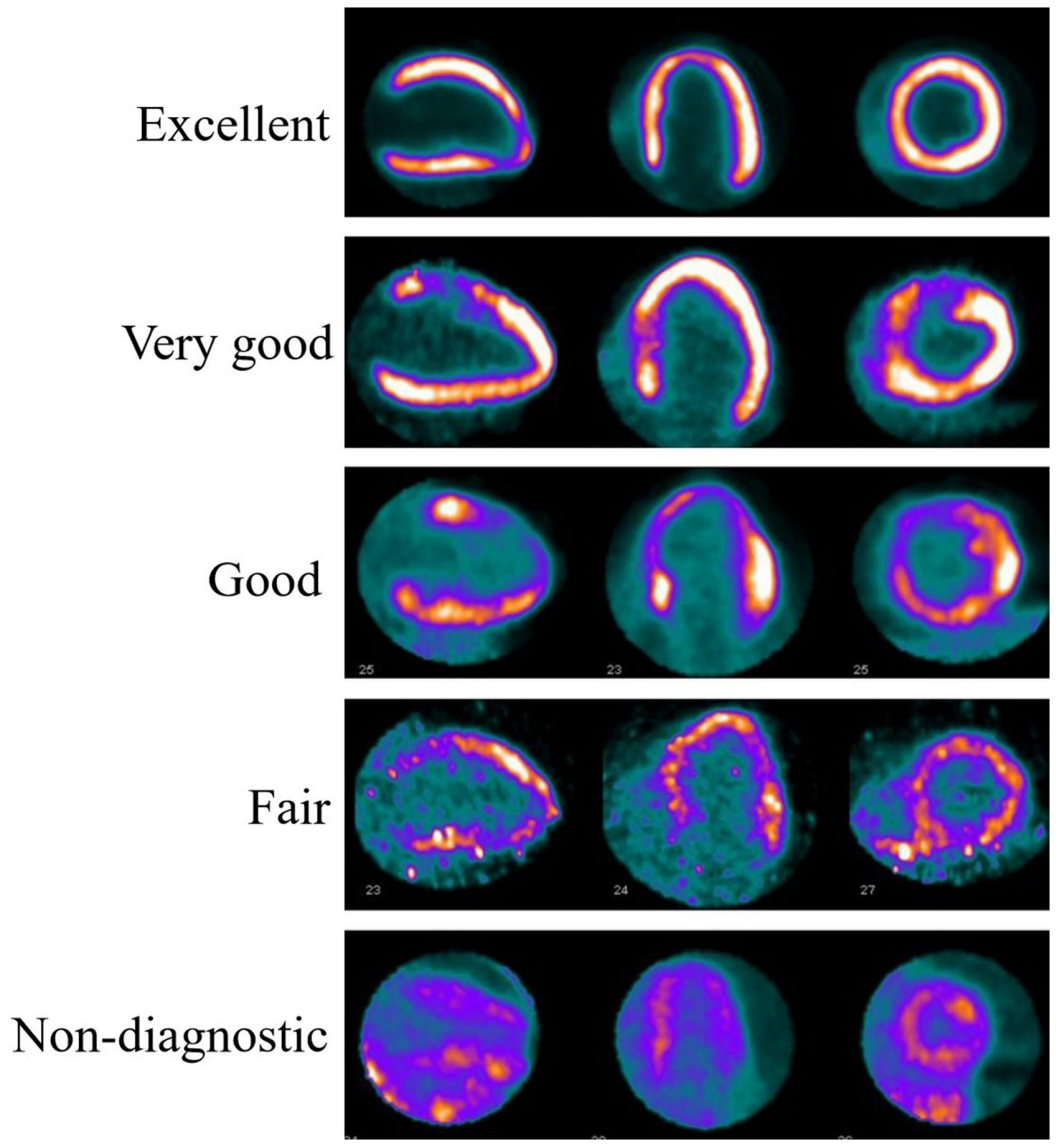
Myocardial ^18^F-FDG image quality was evaluated according to 5-point score system.

To evaluate the reliability for the visual grading of the images, the quality of ^18^F-FDG PET images was evaluated visually by 2 independent experienced nuclear medicine physicians who were unaware of the clinical data. In cases where a disagreement occurred, the discrepancies were resolved by a joint consensus reading. According to the activity of the myocardial ^18^F-FDG uptake and the ^18^F-FDG blood pool activity, cardiac ^18^F-FDG image quality was graded on a 5-point scoring scale (5, excellent; 4, very good; 3, good; 2, fair; and 1, non-diagnostic) (Figure 1). Accordingly, excellent image quality (score 5) was assessed as homogeneous or heterogeneous ^18^F-FDG uptake and no blood-pool activity; very good image quality (score 4) was defined as homogeneous or heterogeneous FDG concentration and mild blood-pool activity; good image quality (score 3) was assessed as homogeneous or heterogeneous ^18^F-FDG signal and moderate blood-pool activity; fair image quality (score 2) was assessed as homogeneous or heterogeneous ^18^F-FDG signal and high blood-pool activity; and non-diagnostic image quality (score 1) denotes that myocardial ^18^F-FDG uptake was either low or absent and high blood-pool activity was visually high. We evaluated inter-observer agreement for the 5-point scoring scale using weighted Cohen’s kappa, and the analysis yielded a kappa value of 0.806 (95% CI: 0.730 ~ 0.882).

### 2.5 Statistical analysis

All statistical analyses were performed using SPSS 26.0 (IBM, United States). Quantitative variables were presented as mean ± SD and qualitative variables are expressed in numbers. The paired t-test was used to compare the quantitative variables, the different groups were compared by ANOVA, and the *χ^2^* test was used to compare the qualitative variables. All *p* value were obtained by two-tailed test, and *p* < 0.05 indicated that the difference was statistically significant.

## 3 Results

### 3.1 Clinical and study characteristics

The characteristics of the study population are summarized in Table 2. For the total study population, baseline blood glucose, peak post-load blood glucose, blood glucose at time of FDG injection, insulin dosage, the number of insulin administration and image quality score were 7.75 ± 1.72 mmol/L, 11.85 ± 1.94 mmol/L, 7.00 ± 1.50 mmol/L, 15.06 ± 9.47 U, 1.94 ± 0.92 times, and 4.43 ± 0.80, respectively. Peak post-load blood glucose level was significantly increased compared with baseline blood glucose (t = −26.106, *p* < 0.001). A significant difference was also found between blood glucose at time of FDG injection and baseline blood glucose level (t = 5.254, *p* < 0.001). Notably, no significant difference in image quality score was found among subgroups categorized by baseline blood glucose levels (*F* = 0.750, *p* = 0.610).

**Table 2:** Patient and study characteristics (mean ± SD).

Baseline blood glucose category	*n*	Age (y)	Sex		Baseline blood glucose	Glucose peak post-load	Blood glucose at time of FDG injection	Dose of Insulin (IU)	Number of insulin administration	Imaging quality scores
			Male	Female						
< = 6	19	62.68 ± 8.71 (42–74)	18	1	5.75 ± 0.12 (5.5–6.0)	11.50 ± 1.19 (9.8–14.1)	6.94 ± 1.80 (4.5–11.0)	12.89 ± 5.84 (6–27)	1.63 ± 0.60 (1–3)	4.42 ± 0.77 (3–5)
6 ~ 7	52	60.02 ± 9.35 (35–80)	49	3	6.46 ± 0.29 (6.1–7.0)	11.13 ± 1.90 (7.1–15.3)	6.24 ± 1.37 (3.7–9.9)	12.23 ± 7.62 (2–36)	1.75 ± 0.76 (1–4)	4.38 ± 0.80 (2–5)
7 ~ 8	43	58.42 ± 12.56 (28–79)	40	3	7.51 ± 0.27 (7.1–8.0)	11.87 ± 1.84 (8.3–17.1)	7.16 ± 1.19 (4.3–9.9)	14.00 ± 8.54 (7–48)	1.91 ± 0.97 (1–4)	4.58 ± 0.66 (2–5)
8 ~ 9	32	62.25 ± 7.34 (46–77)	29	3	8.45 ± 0.28 (8.1–9.0)	12.01 ± 1.90 (9.4–17.5)	7.35 ± 1.26 (5.1–10.1)	15.56 ± 9.71 (7–48)	2.00 ± 0.92 (1–4)	4.34 ± 0.97 (1–5)
9 ~ 10	13	59.92 ± 10.32 (41–74)	12	1	9.37 ± 0.24 (9.1–9.8)	12.98 ± 2.55 (9.6–19.7)	7.64 ± 2.28 (4.5–13.7)	17.38 ± 7.25 (10–31)	2.00 ± 1.08 (1–4)	4.15 ± 1.07 (1–5)
10 ~ 11	13	60.69 ± 7.52 (48–73)	10	3	10.56 ± 0.31 (10.1–11)	12.45 ± 1.34 (10.9–15.3)	7.47 ± 1.44 (4.6–10.0)	19.15 ± 8.49 (8–36)	2.38 ± 0.96 (1–4)	4.46 ± 0.066 (3–5)
>11	7	56.29 ± 11.12 (40–73)	7	0	12.91 ± 1.50 (11.2–14.8)	14.36 ± 1.15 (12.5–16.1)	7.59 ± 1.04 (6.1–8.8)	34.14 ± 15.78 (15–48)	3.14 ± 1.07 (2–4)	4.71 ± 0.49 (4–5)
	179	60.21 ± 9.86 (28–80)	165	14	7.75 ± 1.72 (5.5–14.8)	11.85 ± 1.94 (7.1–19.7)	7.00 ± 1.50 (3.7–13.7)	15.06 ± 9.47 (2–48)	1.94 ± 0.92 (1–4)	4.43 ± 0.80 (1–5)

### 3.2 Imaging results and quality assessment

**Figure 2:**
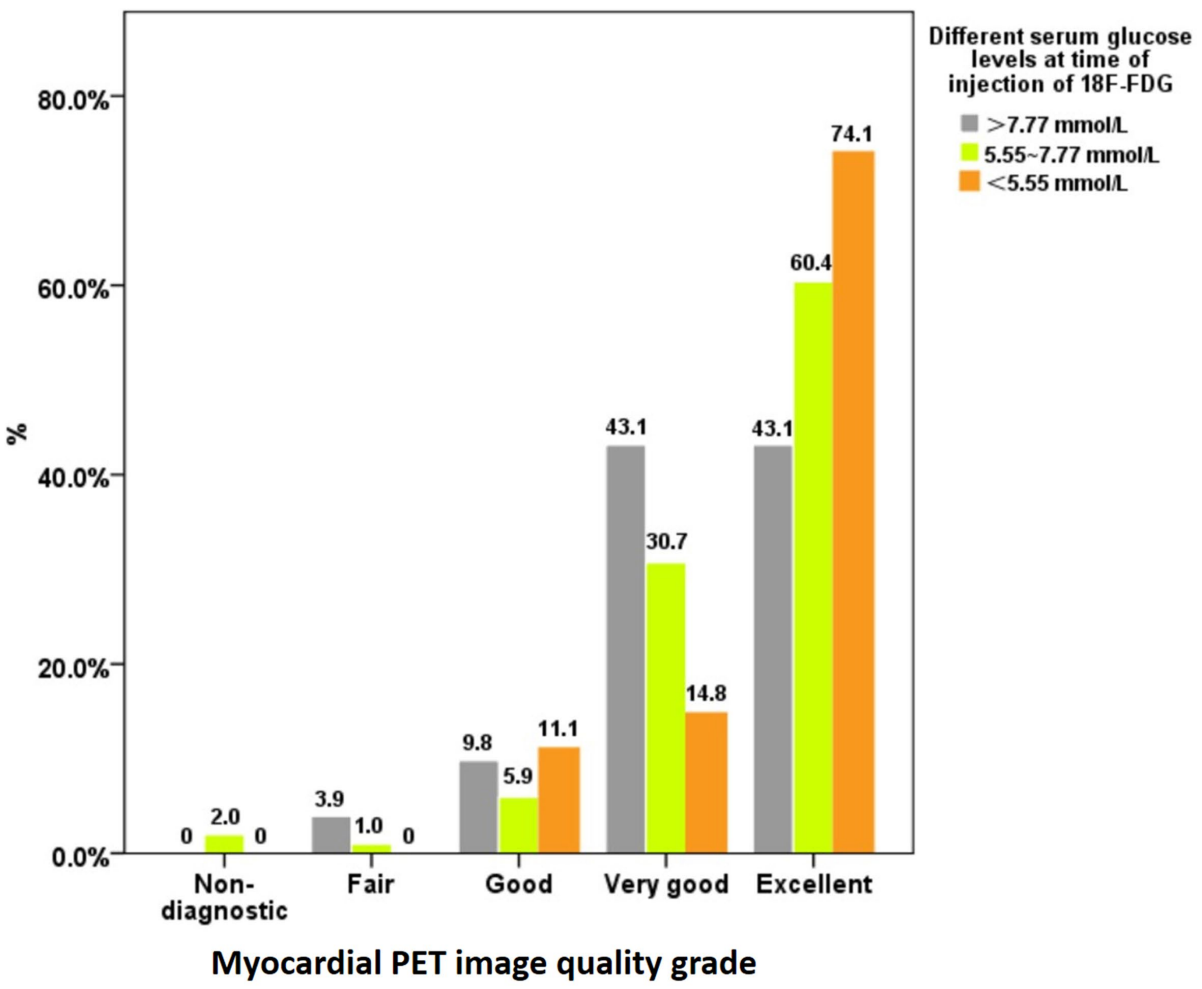
Distribution of myocardial ^18^F-FDG image quality scores when subgrouped and compared between those with different blood glucose at time of FDG injection.

**Figure 3:**
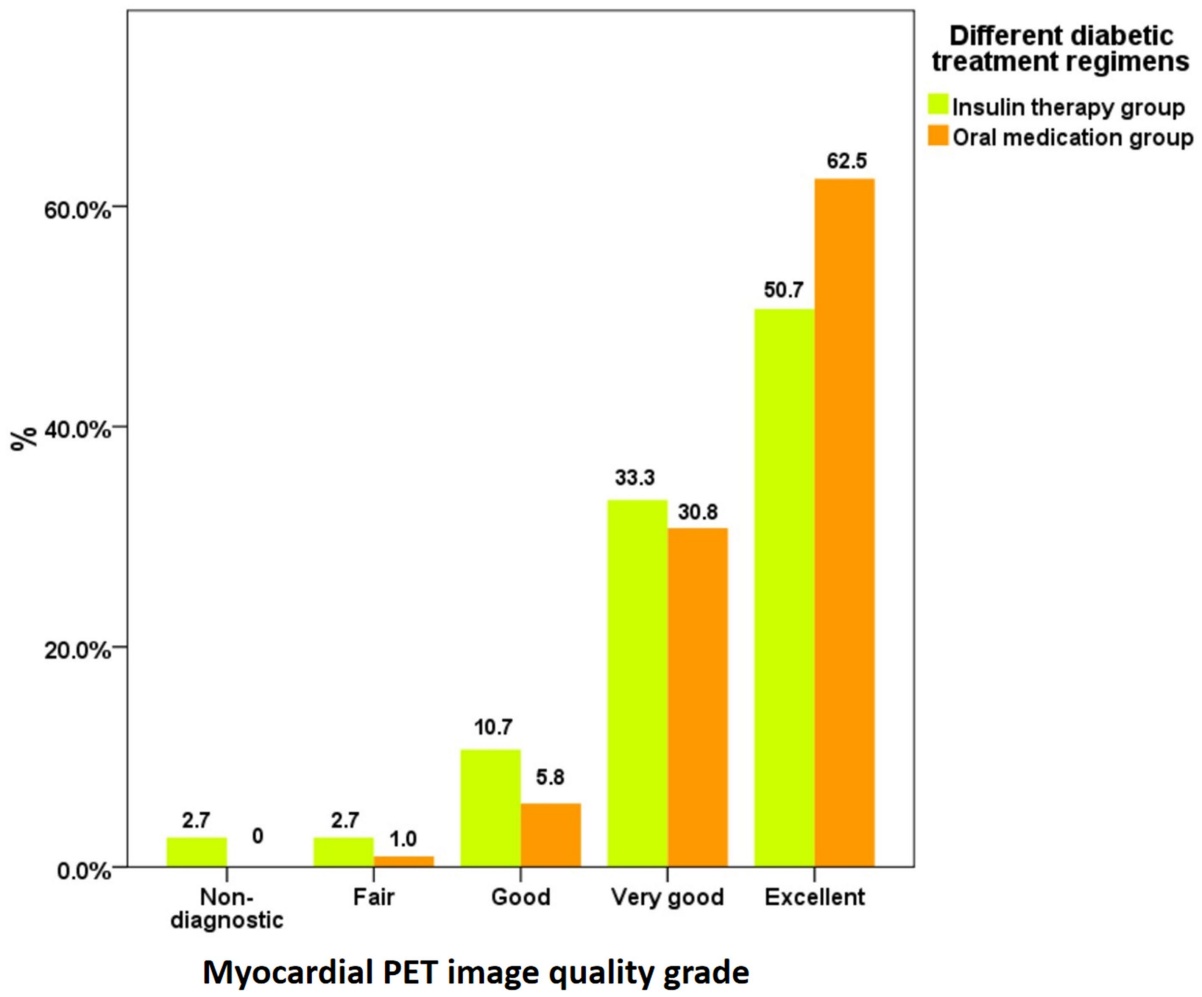
Distribution of myocardial ^18^F-FDG image quality scores when subgrouped and compared between those with different diabetic treatment regimens.

Among the overall study population (*n* = 179), myocardial ^18^F-FDG PET images were scored as excellent in 57.5% (*n* = 103), very good in 31.8% (*n* = 57), good in 7.8% (*n* = 14), fair in 1.7% (*n* = 3), and non-diagnostic in 1.1% (*n* = 2). Patients were stratified into three subgroups according to blood glucose at time of FDG injection: >7.77 mmol/L (*n* = 51), 5.55–7.77 mmol/L (*n* = 101), and <5.55 mmol/L (*n* = 27), the proportions of each image quality grade across the three subgroups were as follows: excellent (43.1%, *n* = 22; 60.4%, *n* = 61; 74.1%, *n* = 20), very good (43.1%, *n* = 22; 30.7%, *n* = 31; 14.8%, *n* = 4), good (9.8%, *n* = 5; 5.9%, *n* = 6; 11.1%, *n* = 3), fair (3.9%, *n* = 2; 1.0%, *n* = 1; 0%, *n* = 0), and non-diagnostic (0%, *n* = 0; 2.0%, *n* = 2; 0%, *n* = 0) (Figure 2). Patients were stratified into two subgroups according to different diabetic treatment regimens: insulin therapy group (*n* = 75) and oral medication group (*n* = 104) the proportions of each image quality grade across the two subgroups were as follows: excellent (50.7%, *n* = 38; 62.5%, *n* = 65), very good (33.3%, *n* = 25; 30.8%, *n* = 32), good (10.7%, *n* = 8; 5.8%, *n* = 6), fair (2.7%, *n* = 2; 1.0%, *n* = 1), and non-diagnostic (2.7%, *n* = 2; 0, *n* = 0) (Figure 3).

**Figure 4:**
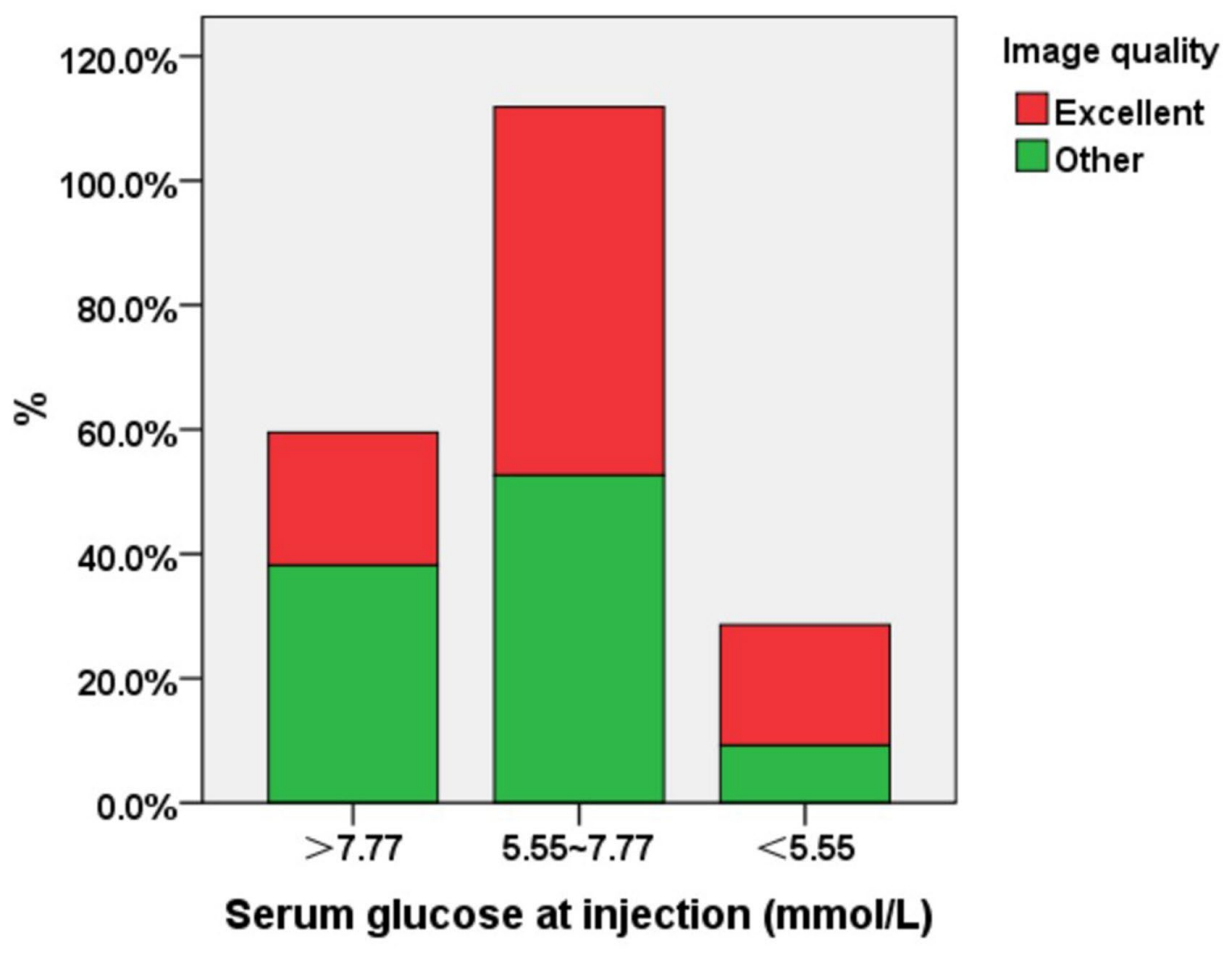
Comparison of image quality among groups stratified by blood glucose at time of FDG injection. Stacked bar chart illustrates the proportions of patients with excellent (red) and “remaining” (green) image quality, categorized by blood glucose at time of FDG injection: >7.77 mmol/L (*n* = 51), 5.55–7.77 mmol/L (*n* = 101), and <5.55 mmol/L (*n* = 27). Percentage values for each category are labeled on the corresponding bars. Patients with blood glucose 5.55–7.77 mmol/L or <5.55 mmol/L at FDG injection exhibited a significantly higher proportion of excellent-quality images than those with glucose levels >7.77 mmol/L.

**Figure 5:**
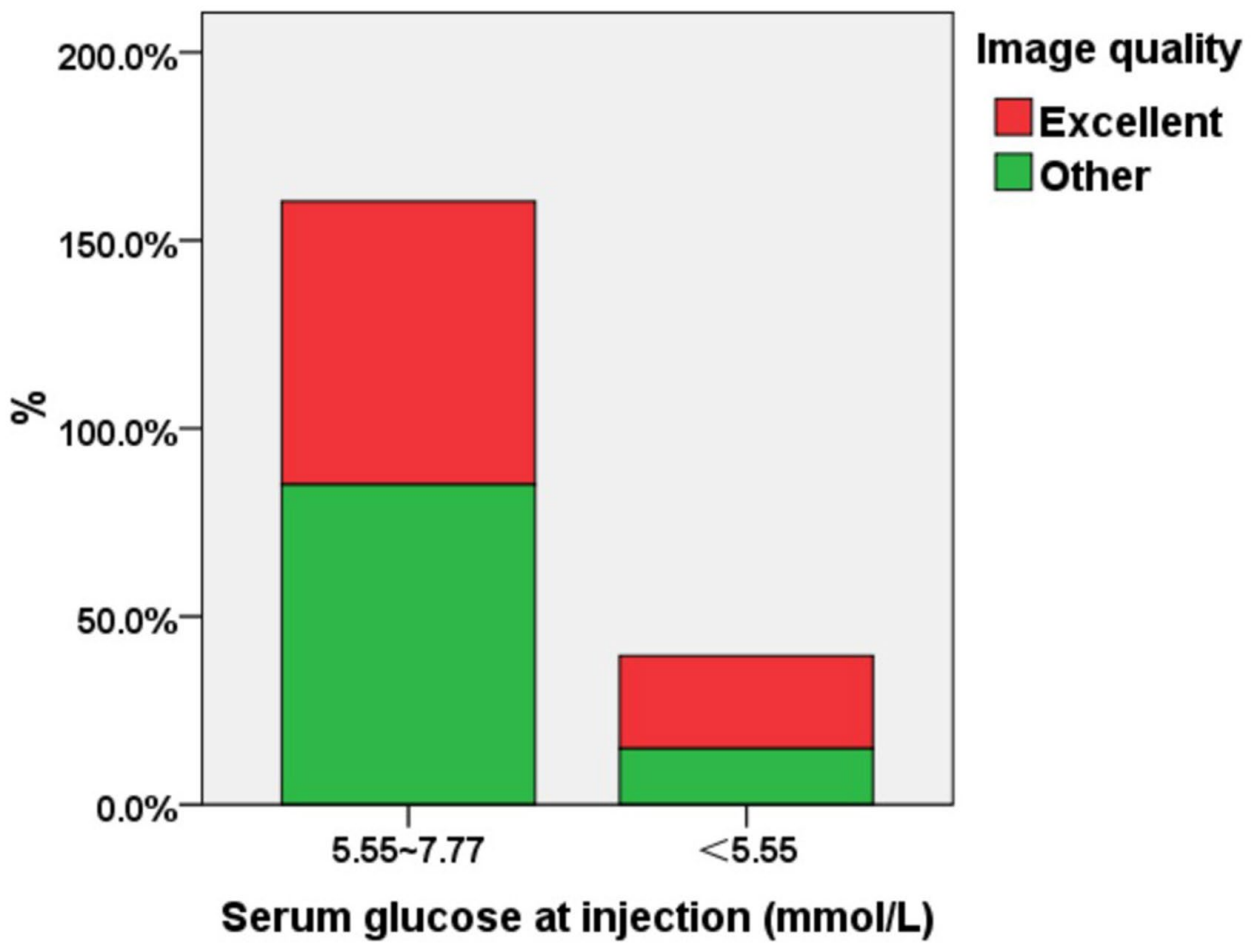
Comparison of image quality between patients stratified by blood glucose at time of FDG injection. Stacked bar chart illustrates the proportions of images graded as excellent (red) and “remaining” (green), including patients with blood glucose at time of FDG injection 5.55–7.77 mmol/L (*n* = 101) and <5.55 mmol/L (*n* = 27). Corresponding percentage values are marked on each bar. No statistically significant differences in image quality were observed between the two subgroups.

Patients were stratified into three subgroups according to blood glucose at time of FDG injection: >7.77 mmol/L (*n* = 51), 5.55–7.77 mmol/L (*n* = 101), and <5.55 mmol/L, to analyze the influence of blood glucose at time of FDG injection on image quality scores. For subgroup comparison, image quality scores were dichotomized: patients scored as “excellent” were compared with the sum of other score group (including very good, good, fair, and non-diagnostic). Patients with blood glucose at time of FDG injection >7.77 mmol/L had a lower rate of excellent image quality relative to those with blood glucose ranging 5.55–7.77 mmol/L and those with blood glucose <5.55 mmol/L (Table 3; Figure 4). However, no statistically significant difference in the rate of excellent images was found between the latter two subgroups (5.55–7.77 mmol/L vs. <5.55 mmol/L) (Table 4; Figure 5).

**Table 3:** Effects of blood glucose at time of FDG injection on image quality scores: a comparison among three groups.

Blood glucose at time of FDG injection (mmol/L)	The number of patients with “excellent” image quality score	The number of patients with “other” image quality score	*n*	*χ^2^*	*p*
>7.77	22 (29.3)	29 (21.7)	51 (51.0)	7.689	0.021
5.55 ~ 7.77	61 (58.1)	40 (42.9)	101 (101.0)		
<5.55	20 (15.5)	7 (11.5)	27 (27.0)		
*n*	103 (103)	76 (76)	179 (179.0)		

**Table 4:** Effects of blood glucose at time of FDG injection on image quality scores: a comparison among two groups.

Blood glucose at time of FDG injection (mmol/L)	The number of patients with “excellent” image quality score	The number of patients with “other” image quality score	*n*	*χ^2^*	*p*
5.55 ~ 7.77	61 (63.9)	40 (37.1)	101 (101)	1.715	0.262
<5.55	20 (17.1)	7 (9.9)	27 (27)		
*n*	81 (81)	47 (47)	128 (128)		

**Figure 6:**
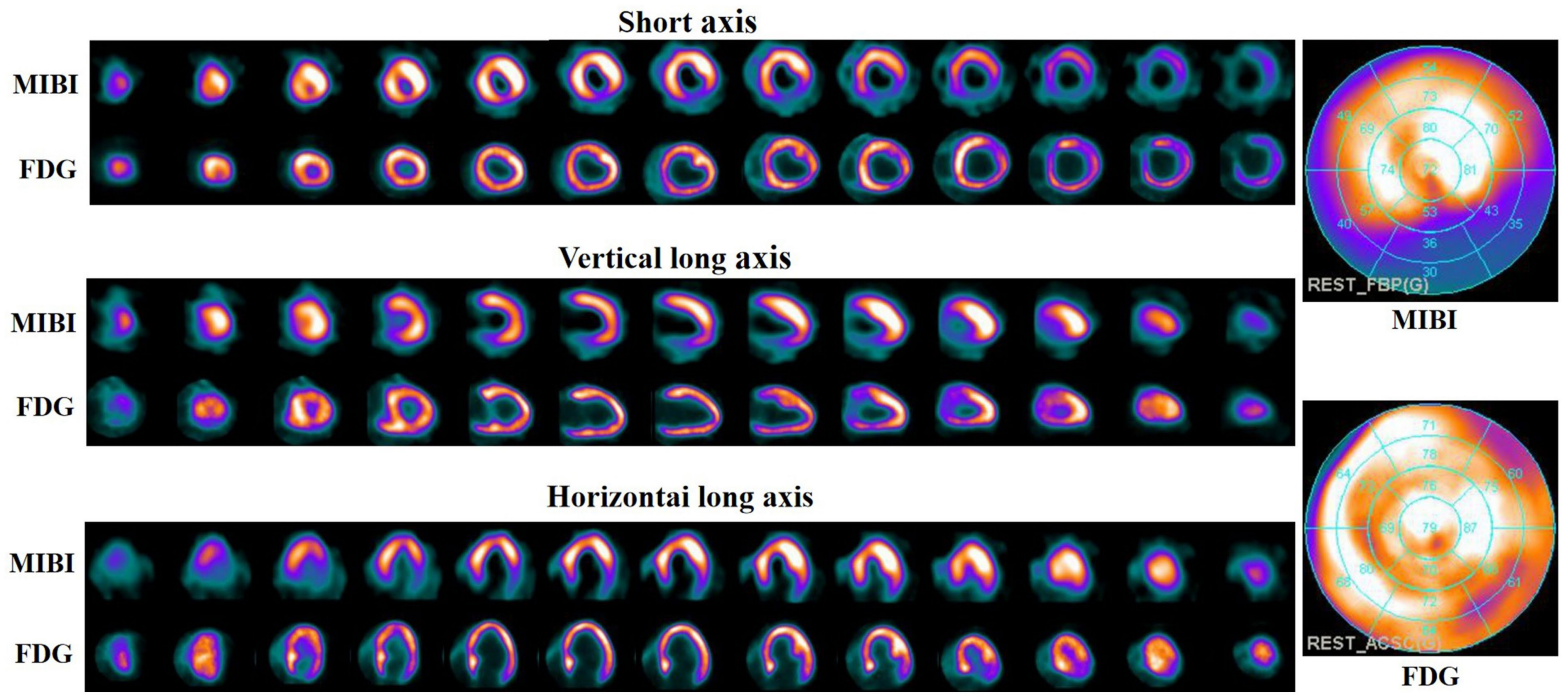
Rest myocardial perfusion ^99m^Tc-MIBI SPECT images demonstrating mismatch with ^18^F-FDG PET images in patient with CHD. SPECT shows moderate hypoperfusion in inferior and inferolateral walls, whereas PET shows normal ^18^F-FDG uptake, consistent with hibernating myocardium.

**Figure 7:**
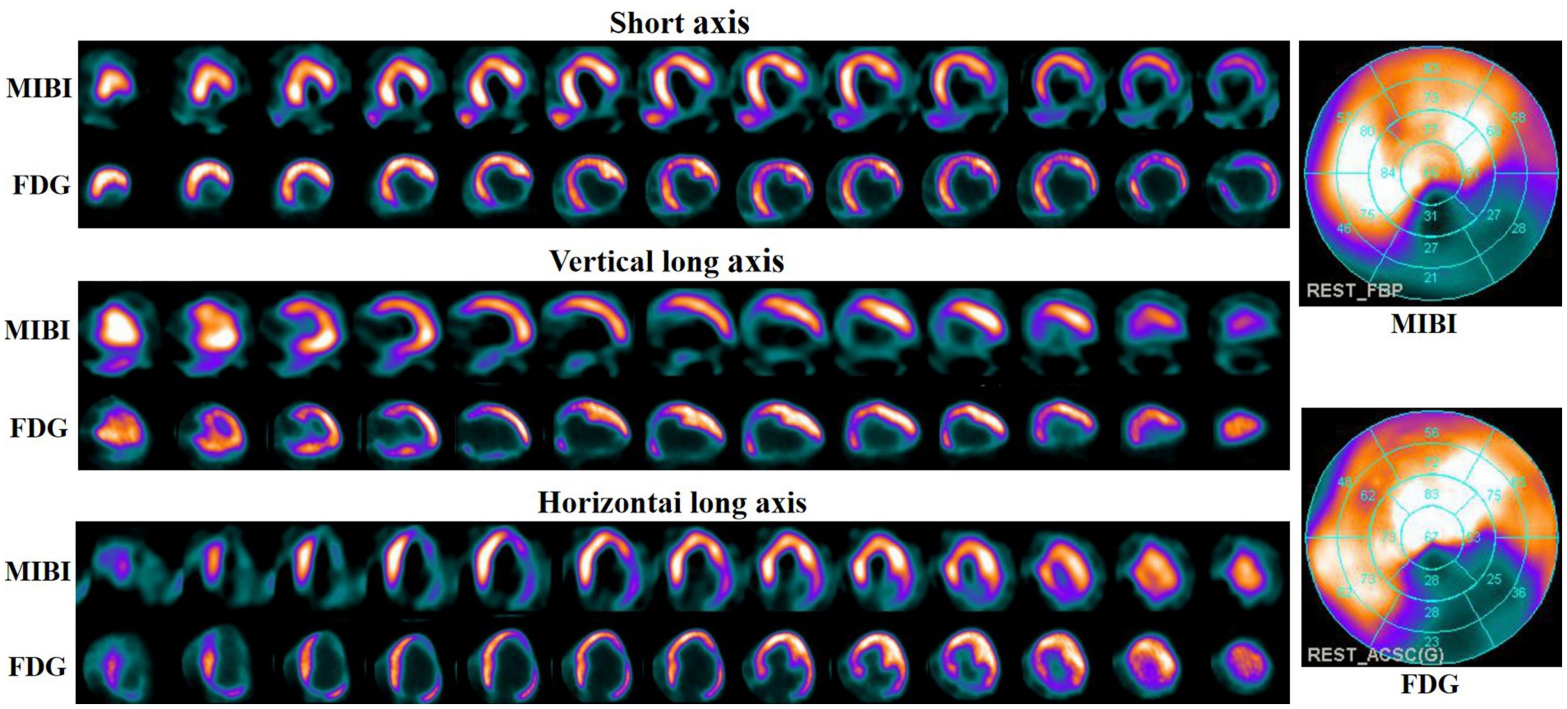
Rest myocardial perfusion ^99m^Tc-MIBI SPECT images demonstrating match with ^18^F-FDG PET/CT images in patient with CHD. SPECT shows severe perfusion defect in inferior, inferolateral walls and apical, and PET shows predominant absence of ^18^F-FDG uptake, thus, findings consistent with scarred myocardium.

Combined analysis of ^99m^Tc-MIBI SPECT and ^18^F-FDG PET demonstrated that among all 179 enrolled patients, 47 patients (26.3%) exhibited complete perfusion–metabolic mismatches (indicating fully viable myocardium, Figure 6), 89 patients (49.7%) showed partial perfusion–metabolic mismatches (indicating partially viable myocardium), and 43 patients (24.0%) had complete perfusion–metabolic matches (indicating scarred myocardium, Figure 7). The total mismatch score in viable myocardium was 19.5 ± 7.7. Stratified by coronary arterial territory, regional mismatch scores were 9.2 ± 6.1 in the left anterior descending artery (LAD) territory, 4.9 ± 5.3 in the left circumflex artery (LCX) territory, and 5.4 ± 4.8 in the right coronary artery (RCA) territory.

### 3.3 Adverse reactions

With the present protocol, supplementary administration of 10 g 50% dextrose after ^18^F-FDG injection was required for safety purposes in only 1 patient (0.6%) who developed asymptomatic hypoglycemia (blood glucose < 3.9 mmol/L); the image quality score of this patient was 4. Besides, five patients reported hunger sensation while maintaining normal blood glucose levels. These patients were permitted to eat approximately 15 min after FDG injection, and their image quality scores ranged from 4 to 5.

## 4 Discussion

The current study establishes a precise, practical protocol involving oral glucose loading and subcutaneous insulin administration prior to ^18^F-FDG injection. This protocol yields good-to-excellent image quality in 97.2% of CAD patients complicated by DM. Notably, blood glucose at time of FDG injection below 5.55 mmol/L also ensures excellent image quality. In addition, the absence of severe adverse reactions across all enrolled patients further validates the safety of this protocol.

As the prevalence of CAD increases, evaluation of myocardial viability is critical for screening candidates who may gain prognostic benefits from revascularization. Numerous studies have verified correlations between myocardial viability detected via cardiac ^18^F-FDG PET and diverse clinical outcomes (10). Glucose serves as one substrate for myocardial energy metabolism. Under normal resting conditions, 60–80% of myocardial energy demand is met by *β*-oxidation of free fatty acids (FFAs), whereas merely 20–40% originates from glucose oxidation and glycolysis (11). However, in cells that are viable but at risk, there is an increase in FDG uptake due to a transition towards anaerobic metabolism and a preference for glucose metabolism over fatty acid metabolism (12). As a radiolabeled glucose analog, ^18^F-FDG PET MMI reflects the glucose uptake and utilization capacity of the myocardium, rendering it one of the most sensitive modalities available for myocardial viability assessment.

Sufficient myocardial ^18^F-FDG uptake serves as a prerequisite for acquiring high-quality images and enabling accurate image interpretation. Myocardial ^18^F-FDG uptake is governed by multiple metabolic factors. In principle, low circulating free fatty acid (FFA) levels together with elevated plasma insulin concentrations facilitate optimal myocardial glucose and ^18^F-FDG uptake. This metabolic milieu can be attained via oral glucose loading to stimulate endogenous insulin secretion, or via combined administration of exogenous insulin and glucose. Multiple metabolic preparation protocols have already been established to improve myocardial ^18^F-FDG uptake, including the hyperinsulinemic-euglycemic clamp technique, oral glucose loading alone (without insulin), intravenous insulin monotherapy (13, 14), oral glucose loading combined with insulin administration, and intravenous glucose loading plus insulin injection (15). Certain researchers also apply fatty acid *β*-oxidation inhibitors such as trimetazidine to elevate myocardial ^18^F-FDG uptake (16). Besides, lipid-lowering agents that lower circulating free fatty acid (FFA) concentrations (e.g., acipimox) are also adopted for preparation prior to myocardial ^18^F-FDG metabolic imaging (17). Nevertheless, despite these optimization strategies, approximately 20–25% of scans still fail to reach diagnostic quality standards, and this unsatisfactory rate is markedly higher among patients with DM (18). The primary cause underlying unsatisfactory ^18^F-FDG PET MMI performance in diabetic patients lies in that diabetic individuals exhibit enhanced myocardial fatty acid uptake, utilization and oxidative stress, accompanied by relatively suppressed myocardial glucose uptake, when compared with non-diabetic counterparts (8, 19–21).

Performing ^18^F-FDG PET MMI in CAD patients complicated by DM remains clinically challenging. Current widely accepted guidelines released jointly by ASNC lack relevant recommendations regarding individualized glucose loading doses customized to different baseline blood glucose levels, nor do they provide separate glucose administration protocols for diabetic versus non-diabetic patients (8). In a former study on ^18^F-FDG PET myocardial metabolic imaging in CAD patients complicated by DM, Mandal et al. (22) stratified patients by a baseline blood glucose cutoff of 10 mmol/L, administering 25 g oral glucose to patients with blood glucose below 10 mmol/L and 12.5 g to those with blood glucose ≥10 mmol/L; however, their protocol lacked further finer stratification of baseline blood glucose ranges. Several prior studies divided participants into five subgroups according to baseline blood glucose and delivered different oral glucose doses correspondingly (13, 23). Nevertheless, their blood glucose stratification enrolled both diabetic and non-diabetic patients rather than being specially designed exclusively for diabetic populations. The present study therefore established a specifical oral glucose loading protocol for diabetic patients undergoing ^18^F-FDG PET MMI. Patients were divided according to baseline blood glucose ranging from ≤6 mmol/L to >11 mmol/L, with precise oral glucose doses ranging from 50 g down to 5 g administered correspondingly, subcutaneous insulin was subsequently administered, and its dosage was adjusted dynamically based on serial blood glucose measurements obtained after glucose loading. Overall imaging quality was favorable: good-to-excellent image quality was attained in 97.2% of participants, while merely 1.1% of patients yielded non-diagnostic images. Satisfactory imaging quality scores were achieved across all baseline blood glucose subgroups, with no statistically significant intergroup differences in image quality detected. Importantly, the imaging quality obtained via our protocol is generally consistent with results reported in earlier investigations (24, 25). The two patients rated with the lowest image quality score (score = 1) both suffered from abdominal obesity, with body mass index (BMI) values of 39.79 and 37.95 kg/m^2^, together with 25-year and 19-year histories of type 2 DM, respectively. Poor imaging outcomes in these two cases were speculated to be linked to severe insulin resistance and markedly elevated serum triglyceride levels.

Although most relevant prior studies employed intravenous insulin protocols (15, 23, 26), the present study selected the subcutaneous insulin protocol due to its merits in convenience and safety. Subcutaneous administration obviates venous cannulation and confers a lower risk of severe hypoglycemia. Admittedly, subcutaneous insulin administration prolongs the MPP. Nevertheless, we implemented a one-day imaging protocol whereby MPI is finished within the MPP window, thereby maintaining favorable patient tolerance.

Current imaging guidelines issued by the ASNC recommend keeping blood glucose at the time of FDG injection within approximately 5.55–7.77 mmol/L to acquire optimal image quality (8). However, certain existing protocols merely require blood glucose to be lower than 140 mg/dL (7.77 mmol/L) before tracer injection, without specifying a lower cutoff limit such as 5.55 mmol/L (27). Our recent study revealed that patients whose blood glucose at time of FDG injection exceeded 7.77 mmol/L were less likely to obtain excellent image quality than those with blood glucose ranging from 5.55 to 7.77 mmol/L and those blood glucose below 5.55 mmol/L. By comparison, no statistically significant difference in the rate of excellent image quality was detected between patients with blood glucose at time of FDG injection maintained at 5.55–7.77 mmol/L and those with blood glucose below 5.55 mmol/L. These results indicate that, in oral glucose loading protocol, proper upward titration of insulin dosage can effectively raise circulating insulin levels and improve myocardial ^18^F-FDG uptake; strict confinement of blood glucose at time of FDG injection to the narrow range of 5.55–7.77 mmol/L is therefore not mandatory.

The side effects observed in this study were similar to those reported in other protocols, mainly including hypoglycemia and hunger (15). Only one patient (0.6%) developed symptomatic hypoglycemia, and five patients reported feelings of hunger. These symptoms resolved immediately after administration of 10 g oral glucose (for hypoglycemia) and consumption of a regular diet at 15 min following FDG injection (for hunger), respectively. The absence of severe adverse reactions in all patients further confirms the favorable safety profile of the proposed protocol.

## 5 Conclusion

A precise and practical protocol consisting of oral glucose loading and subcutaneous insulin administration prior to ^18^F-FDG injection was proven safe and feasibility, yielded good-to-excellent diagnostic image quality in 97.2% of patients with CAD complicated by DM. Blood glucose at time of FDG injection maintained below 5.55 mmol/L also ensured excellent image quality. This protocol holds promise for improving the clinical application and diagnostic efficacy of ^18^F-FDG PET for myocardial viability assessment in routine clinical practice.

## Data Availability

The raw data supporting the conclusions of this article will be made available by the authors, without undue reservation.
